# Genetic characteristics of colostrum refractive index and its use as a proxy for the concentration of immunoglobulins in Holstein cattle

**DOI:** 10.1186/s12711-022-00768-w

**Published:** 2022-12-02

**Authors:** Angela Costa, Giulio Visentin, Arianna Goi, Massimo De Marchi, Mauro Penasa

**Affiliations:** 1 Department of Veterinary Medical Sciences, University of Bologna, Ozzano Dell’Emilia, BO Italy; 2 Department of Agronomy, Food, Natural resources, Animals and Environment, University of Padova, Legnaro, PD Italy

## Abstract

**Background:**

Colostral concentration of immunoglobulins (Ig) is crucial for the passive transfer of antibodies from the cow to the new-born calf. Direct determination of Ig by the gold standard radial immunodiffusion method is demanding in terms of time and costs. For this reason, a refractometer is commonly used at the farm level for an indirect estimation of colostrum quality, which is given as the Ig concentration. In this study, colostrum samples were collected from 548 Italian Holstein cows within 6 h of calving. The refractive index (BRIX, %) of these samples was assessed using a portable optical refractometer, as well as the concentration of total protein, IgG, IgA, and IgM by radial immunodiffusion. A four-trait animal model was used to estimate genetic parameters for BRIX and the different immunoglobulin isotypes. A receiver operating characteristic analysis was carried out to evaluate the BRIX diagnostic accuracy.

**Results:**

Colostral BRIX was moderately heritable (0.26) and its genetic and phenotypic correlations with IgG (0.91, 0.78), IgA (0.57, 0.57), and IgM (0.71, 0.61) were all positive and of similar order, although the genetic correlations were generally higher than the phenotypic correlations. Low-quality colostrum samples, defined as those with an IgG concentration lower than 50 g/L, were accurately identified by the refractive index on the BRIX scale, with an area under the curve of 0.90.

**Conclusions:**

The use of a refractometer is recommended on dairy farms to produce a proxy for colostral Ig concentration. BRIX is a useful phenotyping tool that can be used in cattle to improve the quality of colostrum for first feeding of calves through both traditional genetic and genomic strategies. Improving colostrum quality will reduce the incidence of failure of passive transfer of immunity in young stock.

## Background

Colostrum is the first secretion of the mammary gland after calving [1] and it is essential for the immune protection of the calf since the syndesmochorial placenta of ruminants does not allow the direct transmission of immunoglobulins (Ig) from the dam to the fetus during gestation. Calves are agammaglobulinemic at birth and their ability to develop an effective immune system in early life through passive immunity depends totally on the rapid intake of at least 4 L of good quality colostrum in the first hours of life [2–4].

Immunoglobulins G (IgG), A (IgA), and M (IgM) represent approximately 90%, 5%, and 5% of the total concentration of colostral Ig, respectively [1]. With IgG being the major Ig in the colostrum, it has become the principal component for the evaluation of colostrum [1]. In field conditions, a concentration of IgG lower than 50 g/L is considered indicative of low-quality colostrum [1, 6, 7]. The failure of passive transfer of immunity occurs in calves with an insufficient serum IgG concentration in the first 12 h of life (< 12 g/L) and has been reported to have negative impacts on growth performance, survival, and disease resistance and may affect up to 15% of newborn dairy calves [1, 5].

Radial immunodiffusion (RID) is the gold standard for the determination of Ig concentration in biological fluids such as blood/plasma, saliva, milk, or colostrum in most species. Although it is both a direct and an accurate measurement, its application is limited for on-farm use or when results are needed on a large number of animals [7–9]. Among the currently available indirect methodologies to evaluate colostral Ig, the most popular are those that use indirect portable tools as they can provide a rapid and cost-effective quality assessment [7] within a commercial dairy setting. For instance, refractometers are regularly available to dairy farmers and their measurements are based on the overall matrix density. Such devices determine the total solids content of a liquid/semi-liquid medium by measuring the amount of refracted light, thereby, providing an estimation of the refractive index (BRIX, %) expressed on a BRIX scale. In colostrum, most of the light is refracted by the protein fraction and the correlation between BRIX and the IgG content determined by RID ranges from 0.64 [8] to 0.71 [9] in cattle. Refractometers have several advantages as: (i) they can be used in field conditions, (ii) they do not require trained personnel, and (iii) they are suitable for samples stored in various conditions. In fact, there is evidence that no difference exists in terms of BRIX between fresh and frozen colostrum [9].

A commonly used cut-off for the identification of good quality colostrum corresponds to a BRIX value of 22%, which corresponds to an IgG concentration of approximately 50 g/L. According to Buczinski and Vandeweerd [6], a refractive index lower than 18% is indicative of low-quality colostrum, which is not recommended for first feedings of newborn calves. Moreover, supplementation with good-quality colostrum is recommended in the first hours after birth when colostral BRIX falls within the range of 18 to 22% [6]. Refractometers can also be used to assess with good accuracy the level of antibodies in calf serum [10, 11].

Collection of IgG data on a large scale for colostrum quality phenotyping is very expensive and time-consuming. In addition, colostral composition can vary largely during the first 24 h after calving, which implies that specific and strict sampling protocols are needed to standardize sample collection [12, 13]. According to recent investigations [7, 13, 14], determination of colostrum IgG through infrared spectroscopy is feasible and promising. Near-infrared spectroscopy (NIRS) devices have been shown to be capable of providing accurate predictions in Italian Holstein cows, with a coefficient of determination of 0.83 and a root mean square error of 13.28 g/L in external validation [7]. However, reliable NIRS predictions of IgG can only be obtained through a standardized sampling protocol, the use of representative reference data in the training and validation dataset, and implementation of robust prediction equation(s). Moreover, from a practical viewpoint, the technological advances have not reached a point where implementation cost is sufficiently low for commercial farms. Prediction of colostral IgG through NIRS for breeding and management purposes is promising and can increase the number of phenotypic records accurately with lower implementation costs. Currently worldwide and within Italy, only a limited number of specialist milk laboratories undertaking official routine analyses are equipped with NIRS instruments. This indicates that, currently, acquisition of colostral Ig with NIRS is not technically feasible for mass-phenotyping [13]. Research is in progress to test the prediction ability of mid-infrared tools commonly used for official analyses of milk; preliminary results on diluted colostrum are promising but have not yet undergone validation on a large scale [14–16]. Therefore, evaluation of colostral quality through BRIX has a lower implementation barrier.

In the current study, our aim was to estimate the heritability of BRIX and its genetic correlations with RID-determined Ig isotypes to finally assess the accuracy of refractometer phenotypes as proxies for the target trait to be used for selection purposes.

## Methods

### Experimental design

Because animal interactions occurred only between the farmers and their own animals, ethical approval for this study was not required as per institutional or local legislation, however, informed consent was obtained from the farmers enrolled within the study. Before the start of the trial, the sampling protocol was provided and explained to each farmer, highlighting the study aims and methodology. According to the experimental design, only the first colostrum had to be sampled for the study; therefore, the calf was separated from the dam as soon as possible after birth and was not allowed to suckle. Colostrum from unsupervised calvings was not collected for the study.

The sampling procedure of this study is the same as that described in [12, 13]; briefly, collection of individual colostrum samples (n = 548) took place on nine commercial farms of northern Italy from 2019 to 2020, covering the four calendar seasons. Farms involved in the study had between 60 and 190 lactating Holstein cows and used a total mixed ration method to feed animals, free stall barns, twice-a-day milking, with access to pasture. Moreover, none of the enrolled farms underwent a vaccination protocol against rotavirus, coronavirus, or E. coli. To reduce the impact of time on colostrum composition, only colostrum samples collected within 6 h after calving were included in the study. A representative colostrum sample was taken from a cow based on the pooled colostrum from all functional quarters at first milking.

For each cow, 120 mL were collected in a single sterile plastic tube without preservative (SMIPA srl, Vicenza, Italy). Farmers were in charge of colostrum collection and were instructed to annotate the cow ID on the tube and to freeze (− 20 °C) the samples as soon as possible. Periodically, colostrum samples were retrieved from the farms, transferred to the laboratory of the Department of Agronomy, Food, Natural resources, Animals and Environment of the University of Padova (Legnaro, Italy), and stored at − 20 °C until analysis. Date of birth, date of calving, and parity of sampled cows were retrieved and farm-specific general information about colostrum management was registered.

### Quantification of Ig and BRIX

Samples were thawed overnight at 4 °C in a waterbath and RID analyses were carried out using bovine-specific assays [12]. For this study, 34 ‘Bovine IgG RID Kit’, 34 ‘Bovine IgM RID Kit’, and 30 ‘Bovine IgA RID Kit’ were purchased in advance from Triple J Farms (Bellingham, WA, US). Each kit included a plate and reference sera. Dilution in pure deionized water 1:5 (v/v) for IgG and 1:3 (v/v) for IgA and IgM was performed to reduce the concentration of the target component in such a way that the concentration would fall within the assay detection range [12]. Five µL of diluted colostrum were subsequently pipetted in the wells of the three RID plates (IgG, IgA, and IgM, respectively). After incubation at 20 °C for 24 h, plates were scanned at high resolution to measure the diameter of precipitated rings using the image processing program ImageJ (Laboratory for Optical and Computational Instrumentation, University of Wisconsin-Madison, WI). For each well, the precipitated ring was measured in duplicate to calculate the final diameter (mm) as the average of the two measurements. The final diameter was used to calculate the concentration (g/L) of the target component (IgG, IgA, or IgM) by using the known concentrations and measured diameter of the three reference sera to calculate a standard curve for each individual plate. Concentrations of the reference sera for each Ig were: 1.80, 14.72, and 28.03 g/L for IgG, 0.53, 1.94, and 3.87 g/L for IgA, and 0.62, 2.00, and 3.81 g/L for IgM.

Non-readable or non-circular RID rings due to issues during colostrum dilution or pipetting were treated as missing values, and the same rule was applied to samples with concentration values that were beyond the detection range of the kit. Repeatability of this method was preliminarily tested in cow colostrum as described by [12] and [13]. Based on the indications given by the US Department of Health and Human Services, Food and Drug Administration [16], the repeatability of RID, measured through the intra-assay coefficient of variation, was sufficiently robust (< 10%) to refrain from duplicate analyses [12, 13]. For the duration of the trial, RID analyses were carried out by the same laboratory technician, to reduce interpersonal variation.

Colostral BRIX values were measured using an optical refractometer manufactured by Euro Horse Line (San Marino) with a working range from 0 to 32%. After tube inversion, 0.25 mL of colostrum were placed on the daylight plate of the device and the BRIX values were recorded in duplicate for each sample by the same operator for the duration of the study. The average of the two measurements was used in later analyses.

A small representative aliquot of colostrum was lyophilized through a freeze-drying process and subsequently analysed with the Kjeldhal method according to AOAC [17] in order to determine the total protein content (PC_C_, %). For each cow for which the information was available, the milk protein content (PC_M_, %) and yield (PY_M_, kg/d) at the first test-day, i.e., between 5 to 75 days in milk, were retrieved from the national database of Italian Holstein, Brown Swiss and Jersey Association (ANAFIBJ, Cremona, Italy). After merging the available test-day milk data and colostrum traits, information on 500 out of the 548 cows was available for further analyses.

### Data analysis

The trial was designed to ensure that only one sample per cow was present in the final dataset. Prior to the multi-trait genetic analysis, significance of the fixed effects of parity (1, 2, 3, 4, and ≥ 5), calving season (January–March, April–June, July–September, and October–December), calving year (2019 and 2020), and herd (n = 9), and least squares means of BRIX for these effects were tested using the software ASReml v4.1 [18]. The model also included random additive genetic animal effects $\sim N(0, \mathbf{A}{\sigma }_{\text{ag}}^{2}$) and a residual $\sim N(0, \mathbf{I}{\sigma }_{\text{e}}^{2})$, where $\mathbf{A}$ is the additive genetic relationship matrix, ${{\sigma}_{\text{ag}}^{2}}$ is the additive genetic variance, $\mathbf{I}$ is an identity matrix of appropriate order, and ${{\sigma}_{\text{e}}^{2}}$ is the residual variance. The pedigree file (n = 6714 individuals) used for setting up $\mathbf{A}$ included all sampled cows and their ancestors up to six generations back.

Subsequently, a four-trait animal model was run with the same software [18] to estimate genetic parameters for BRIX, IgG, IgA, and IgM using the aforementioned fixed and random effects. In matrix notation, the model can be represented as:

1 $$\left[\begin{array}{c}\begin{array}{c}{\mathbf{y}}_{\mathbf{1}}\\ \dots \end{array}\\ {\mathbf{y}}_{\mathbf{4}}\end{array}\right]=\left[\begin{array}{ccc}{\mathbf{\mathbf{X}}}_{\mathbf{1}}& \mathbf{0}& \mathbf{0}\\ \mathbf{0}& \ddots & \mathbf{0}\\ \mathbf{0}& \mathbf{0}& {\mathbf{X}}_{\mathbf{4}}\end{array}\right]\left[\begin{array}{c}{\mathbf{b}}_{\mathbf{1}}\\ \dots \\ {\mathbf{b}}_{\mathbf{4}}\end{array}\right]\text{+}\left[\begin{array}{ccc}{\mathbf{Z}}_{\mathbf{1}}& \mathbf{0}& \mathbf{0}\\ \mathbf{0}& \ddots & \mathbf{0}\\ \mathbf{0}& \mathbf{0}& {\mathbf{Z}}_{\mathbf{4}}\end{array}\right]\left[\begin{array}{c}{\mathbf{a}}_{\mathbf{1}}\\ \dots \\ {\mathbf{a}}_{\mathbf{4}}\end{array}\right]\text{+}\left[\begin{array}{c}{\mathbf{e}}_{\mathbf{1}}\\ \dots \\ {\mathbf{e}}_{\mathbf{4}}\end{array}\right],$$

where ${\mathbf{y}}_{\mathbf{1}}$, ${\mathbf{y}}_{\mathbf{2}}$, ${\mathbf{y}}_{\mathbf{3}}$, and ${\mathbf{y}}_{\mathbf{4}}$ are the vectors of phenotypic observations of the dependent variables BRIX, IgG, IgA, and IgM, respectively; $\mathbf{b}$ is the vector of fixed effects; $\mathbf{a}$ is the vector of random additive genetic effects; $\mathbf{e}$ is the vector of random residuals; and $\mathbf{X}$ and $\mathbf{Z}$ are incidence matrices relating the corresponding effects to the dependent variable. In the four-trait animal model, the variance–covariance structure of the random effects was assumed equal to:

2 $$\mathbf{var}\left[\begin{array}{l}{\mathbf{a}}_{\mathbf{1}}\\ \dots \\ \begin{array}{l}{\mathbf{a}}_{\mathbf{4}}\\ {\mathbf{e}}_{\mathbf{1}}\\ \begin{array}{l}\dots \\ {\mathbf{e}}_{\mathbf{4}}\end{array}\end{array}\end{array}\right]=\left[\begin{array}{l}\begin{array}{llllll}\mathbf{A}{{\upsigma}}_{{{\text{a}}{\text{g}}}_{1}}^{2}& \cdots & \mathbf{A}{{\upsigma}}_{{{\text{ag}}}_{14}} & \mathbf{0}& \mathbf{0}& \mathbf{0}\\ \vdots & \ddots & \vdots & \mathbf{0}& \mathbf{0}&\mathbf{0} \\ \mathbf{A}{{\upsigma}}_{{{\text{ag}}}_{14}}& \cdots & \mathbf{A}{{\upsigma}}_{{\text{ag}}_{{\text{4}}}}^{2} & \mathbf{0}& \mathbf{0}& \mathbf{0} \\ \mathbf{0}& \mathbf{0}& \mathbf{0}& \mathbf{I}{{\upsigma}}_{{{\text{e}}}_{1}}^{2}& \cdots & \mathbf{I}{\upsigma}_{{\text{e}}_{14}} \\ \mathbf{0}& \mathbf{0}&\mathbf{0}& \vdots & \ddots & \vdots \\ \mathbf{0}& \mathbf{0}& \mathbf{0}& \mathbf{I}{\upsigma}_{{\text{e}}_{14}}& \cdots & \mathbf{I}{\upsigma}_{{\text{e}}_{4}}^{2}\end{array}\end{array}\right].$$

The phenotypic variance (${{{\upsigma}}}_{{\text{p}}}^{2}$), heritability (${{{h}}}^{2}$), coefficient of phenotypic (${\mathrm{CV}}_{\mathrm{p}}$) and additive genetic variation (CV_ag_), and the phenotypic (r_p_) and additive genetic correlations (r_ag_) were calculated by means of conventional formulas [18]. To test if ${{{h}}}^{2}$ estimates differed significantly from 0, the log-likelihood ratio test was used [19]. Briefly, the log-likelihood of the control model was compared to a model where the additive genetic variance was constrained to be approximately zero (= 1 $\times$ 10^–3^). The significance threshold was set at P < 0.05.

The r_p_ and r_ag_ of BRIX and IgG with PC_C,_ PC_M_, and PY_M_ were also estimated using the approach described above. Two multivariate models, one with colostral BRIX [Eq. (3)] and another one with colostral IgG [Eq. (4)], were run including the same fixed and random effects described for Eq. (1) and using the following phenotypic vectors $\mathbf{y}$:

3 $$\left[\begin{array}{c}\begin{array}{c}{\mathbf{y}}_{\mathbf{B}\mathbf{R}\mathbf{I}\mathbf{X}}\\ {\mathbf{y}}_{{\mathbf{P}\mathbf{C}}_{\mathbf{C}}}\end{array}\\ \begin{array}{c}{\mathbf{y}}_{{\mathbf{P}\mathbf{C}}_{\mathbf{M}}}\\ {\mathbf{y}}_{{\mathbf{P}\mathbf{Y}}_{\mathbf{M}}}\end{array}\end{array}\right],$$

and

4 $$\left[\begin{array}{c}\begin{array}{c}{\mathbf{y}}_{\mathbf{I}\mathbf{g}\mathbf{G}}\\ {\mathbf{y}}_{{\mathbf{P}\mathbf{C}}_{\mathbf{C}}}\end{array}\\ \begin{array}{c}{\mathbf{y}}_{{\mathbf{P}\mathbf{C}}_{\mathbf{M}}}\\ {\mathbf{y}}_{{\mathbf{P}\mathbf{Y}}_{\mathbf{M}}}\end{array}\end{array}\right],$$

where ${\mathbf{y}}_{\mathbf{B}\mathbf{R}\mathbf{I}\mathbf{X}}$, ${\mathbf{y}}_{{\mathbf{P}\mathbf{C}}_{\mathbf{C}}}$, ${\mathbf{y}}_{{\mathbf{P}\mathbf{C}}_{\mathbf{M}}}$, ${\mathbf{y}}_{{\mathbf{P}\mathbf{Y}}_{\mathbf{M}}}$ and ${\mathbf{y}}_{\mathbf{I}\mathbf{g}\mathbf{G}}$ contain phenotypic observations available for BRIX, PC_C_, PC_M_, PY_M_, and IgG, respectively. The use of two separate analyses was needed to avoid convergence issues, which usually arise with the REML algorithm when performing multivariate models to estimate unique solutions and (co)variance components for two (or more) traits that are strongly correlated, such as BRIX and IgG.

### Refractive index cut-off

The R package ‘OptimalCutpoints’ with the Youden method [20, 21] was used to calculate the BRIX value, which is useful to discriminate good- from low-quality colostrum samples, by simultaneously reducing specificity (true negative cases) and improving sensitivity (true positive cases). Following Šimundić [22], the BRIX classification accuracy was evaluated by the area under the curve (AUC) obtained. Receiver operating characteristic (ROC) analyses were carried out considering a RID IgG threshold at 50 and 60 g/L.

## Results

**Fig. 1:**
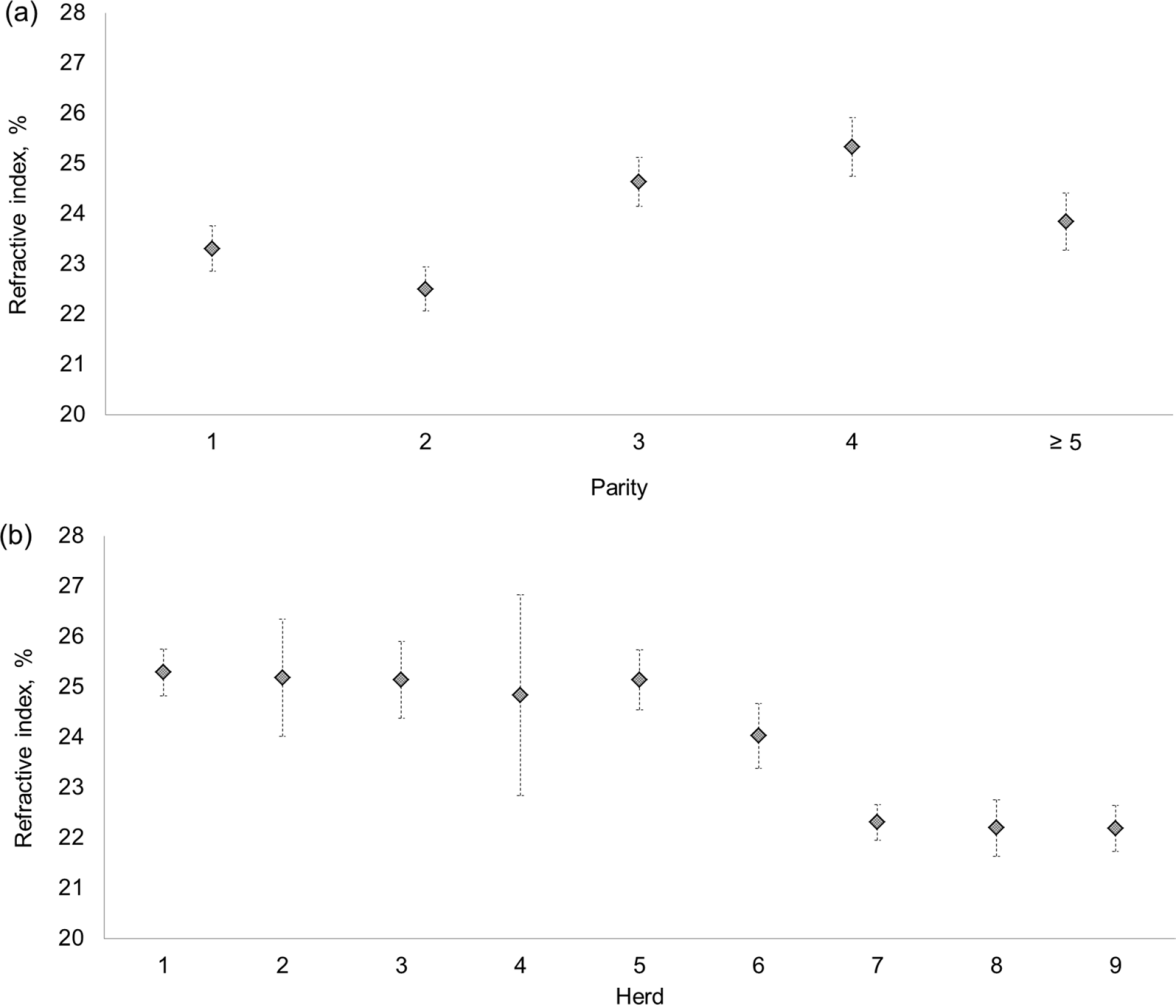
Least squares means of the colostrum refractive index. Fixed effects of (**a**) parity (P < 0.001) and (**b**) herd (P < 0.001) are presented

Descriptive statistics of the traits investigated in the present study are in Table 1. The colostrum BRIX averaged 23.19% with a standard deviation of 4.17% and the three Ig differed in terms of average concentration. Large differences were observed between the minimum and maximum values of Ig isotypes (Table 1), with the range being 206.54 (IgG), 15.45 (IgA), and 13.75 g/L (IgM). Overall, the ${\mathrm{CV}}_{\mathrm{ag}}$ was nearly half the ${\mathrm{CV}}_{\mathrm{p}}$ (Table 1) and the most variable trait at the phenotypic level was IgA with a ${\mathrm{CV}}_{\mathrm{p}}$ of 0.59. The trait with the largest genetic variation was IgM. Colostral BRIX differed across parities and herds (P < 0.001), while it was similar across calving seasons (P = 0.247) and years (P = 0.400). The BRIX estimates of older cows (parities 3, 4, and ≥ 5) were larger than those of younger cows (parities 1 and 2; Fig. 1). In fact, the least squares means ranged from 22.50 ± 0.43% (parity 2) to 25.33 ± 0.58% (parity 4). Regarding the herds, BRIX estimates ranged from 22.19 ± 0.46% to 25.29 ± 0.46%.

**Table 1:** Descriptive statistics and heritability (standard error) of colostrum traits

Trait	n	Mean	Minimum	Maximum	${\mathrm{CV}}_{\mathrm{p}}$	${\mathrm{CV}}_{\mathrm{ag}}$	${\sigma }_{\mathrm{p}}^{2}$	${h}^{2}$
BRIX, %	542	23.19	10.40	32.00	0.18	0.09	16.04	0.26 (0.14)
IgG, g/L	494	92.71	1.96	208.50	0.39	0.16	1103.10	0.21 (0.14)
IgA, g/L	423	4.84	0.13	15.58	0.59	0.22	6.51	0.17 (0.16)
IgM, g/L	481	5.17	0.26	14.01	0.49	0.24	5.87	0.27 (0.15)

*CV*_*p*_ coefficient of phenotypic variation calculated as the ratio of unadjusted phenotypic standard deviation (raw data) to the overall mean, *CV*_*ag*_ coefficient of additive genetic variation calculated as the ratio of additive genetic standard deviation to the overall mean, ${\sigma }_{p}^{2}$ , phenotypic variance, calculated as the sum of the additive genetic and residual variances,* h*^2^ heritability, *BRIX*  refractive index, *IgG*  immunoglobulins G, *IgA*  immunoglobulins A, *IgM*  immunoglobulins M

The ${h}^{2}$ of BRIX was 0.26 ± 0.14, which is slightly higher than that of IgG (0.21 ± 0.14; Table 1) and the least and most heritable traits were IgA (0.17 ± 0.16) and IgM (0.27 ± 0.15). The three Ig isotypes were positively correlated with each other and also had a strong and positive association with BRIX (Table 2). The IgG fraction had the strongest correlation to BRIX both phenotypically (0.78 ± 0.02) and genetically (0.91 ± 0.14). Excluding the ${\mathrm{r}}_{\mathrm{p}}$ and ${\mathrm{r}}_{\mathrm{ag}}$ between BRIX and the other Ig isotypes, the strongest genetic and phenotypic correlations were estimated between IgG and IgM. On the contrary, the weakest ${\mathrm{r}}_{\mathrm{p}}$ was estimated between IgA and IgM (0.55 ± 0.04), and the weakest ${\mathrm{r}}_{\mathrm{ag}}$ were between IgA, and both IgG and IgM (Table 2). The ${\mathrm{r}}_{\mathrm{ag}}$ of the present study were characterized by relatively large standard errors (Tables 1 and 2); in particular, the largest standard error was estimated for the ${\mathrm{r}}_{\mathrm{ag}}$ between IgG and IgA and was equal to 0.46 (Table 2).

**Table 2:** Additive genetic (above the diagonal) and phenotypic (below the diagonal) correlations estimated between the colostrum traits

Trait	BRIX	IgG	IgA	IgM
BRIX, %	–	0.91 (0.14)	0.57 (0.35)	0.71 (0.23)
IgG, g/L	0.78 (0.02)	–	0.39 (0.46)	0.49 (0.31)
IgA, g/L	0.57 (0.03)	0.56 (0.04)	–	0.39 (0.41)
IgM, g/L	0.61 (0.03)	0.61 (0.03)	0.55 (0.04)	–

*BRIX*  refractive index, *IgG*  immunoglobulins G, *IgA*  immunoglobulins A, *IgM*  immunoglobulins M

Standard errors are within parentheses

Mean values for PC_C_, PC_M_, and PY_M_ were 14.46 ± 3.71%, 3.19 ± 0.36%, and 1.21 ± 0.29 kg/d, respectively, and ${h}^{2}$ from the four-trait analysis including BRIX [Eq. (3)] were 0.27 ± 0.14, 0.09 ± 0.11, and 0.03 ± 0.11, respectively. These estimates were similar to those obtained in the four-trait analysis including IgG [Eq. (4)]; 0.27 ± 0.14, 0.07 ± 0.11, and 0.03 ± 0.11]. The correlations of BRIX with PC_C_, PC_M_, and PY_M_ were similar to those of IgG with the same traits (Table 3), and the ${\mathrm{r}}_{\mathrm{p}}$ and ${\mathrm{r}}_{\mathrm{ag}}$ between PC_C_ and PC_M_ were weak, averaging − 0.14 (${\mathrm{r}}_{\mathrm{ag}}$) and 0.03 (${\mathrm{r}}_{\mathrm{p}}$, results not shown).

**Table 3:** Additive genetic (${\mathrm{r}}_{\mathrm{ag}}$) and phenotypic (${\mathrm{r}}_{\mathrm{p}}$) correlations of protein content and protein yield with BRIX and IgG

Trait	BRIX, %		IgG, g/L	
	${\mathrm{r}}_{\mathrm{ag}}$	${\mathrm{r}}_{\mathrm{p}}$	${\mathrm{r}}_{\mathrm{ag}}$	${\mathrm{r}}_{\mathrm{p}}$
PC_C_, %	0.99 (0.04)	0.92 (0.01)	0.92 (0.08)	0.85 (0.08)
PC_M_, %	− 0.15 (0.51)	0.03 (0.05)	− 0.04 (0.56)	0.01 (0.05)
PY_M_, kg/d	− 0.03 (0.60)	0.02 (0.05)	0.09 (0.66)	− 0.09 (0.05)

*BRIX*  refractive index, *IgG*  immunoglobulins G, *PC*_*C*_  colostrum (total) protein content, *PC*_*M*_  milk (total) protein content, *PY*_*M*_  milk protein yield

Standard errors are within parentheses

**Fig. 2:**
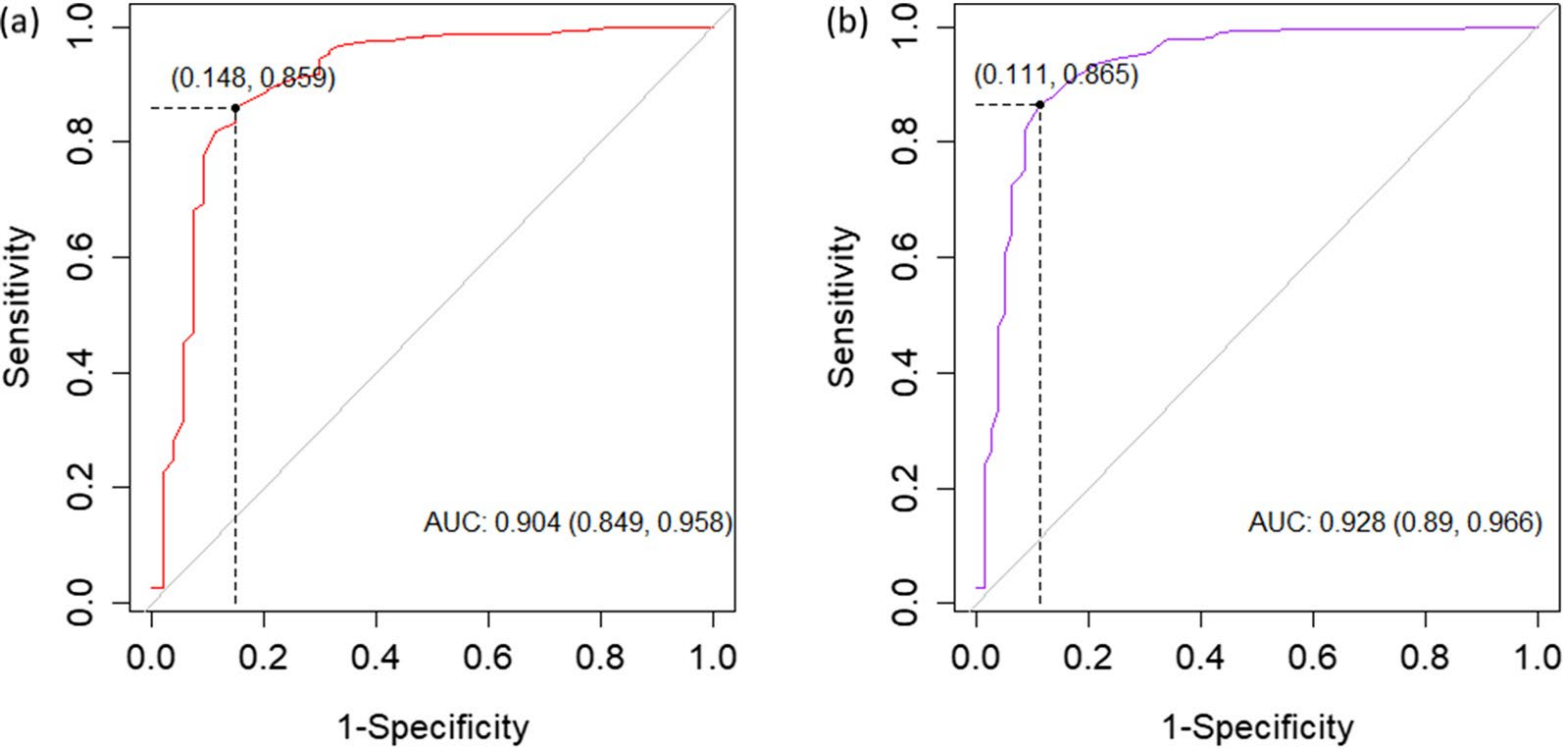
Graphical output of receiver operating characteristic analyses for the colostrum refractive index discriminant ability. The ability to discriminate good- and low-quality colostrum samples was tested considering (**a**) 50 and (**b**) 60 g/L of immunoglobulins G as a threshold. Coordinates of the optimal cut-off (●) are specified along with the area under curve (AUC) with 95% confidence interval

A summary of the output of the ROC analyses is in Table 4 and a graphical representation of curves is in Fig. 2. The analyses revealed that the best specificity and sensitivity were at a BRIX cut-off of 20.2 and 21.0% for the two IgG thresholds considered (Table 4). The AUC, representing the diagnostic accuracy calculated through the ROC analysis, was 0.90 for the threshold at 50 g/L of IgG and 0.93 for the threshold at 60 g/L of IgG.

**Table 4:** Summary of the receiver operating characteristic analyses carried out for the colostrum refractive index (BRIX, %)

Parameter	Threshold at 50 g/L		Threshold at 60 g/L	
	Estimate	95% confidence interval	Estimate	95% confidence interval
BRIX cut-off	20.2		21.0	
Specificity	0.85	(0.73, 0.92)	0.89	(0.80, 0.94)
Sensitivity	0.86	(0.82, 0.89)	0.87	(0.83, 0.90)
Positive predictive value	0.98	(0.96, 0.99)	0.98	(0.95, 0.99)
Negative predictive value	0.43	(0.34, 0.53)	0.57	(0.48, 0.65)

## Discussion

The average BRIX value (23.19%; Table 1) observed in the present study was consistent with other results reported in Holstein [9, 23] and Norwegian Red [24] cattle. In general, small differences across different studies are attributable to differences in experimental design and sampling protocol. Researchers that measured cow colostrum IgG via RID found mean values equal to 73.4 ± 26.2 [23] and 56.8 ± 26.9 g/L [24], which are slightly lower than those observed in the present study (Table 1). Nevertheless, our range of values is similar to that of Johnsen et al. [25], i.e. 5 to 129 g/L. In a study carried out on Canadian beef cows [26], more than 75% of the samples (n = 416) were collected within one hour from parturition, the moment where the IgG release in colostrum is maximum. This explains why average RID IgG was greater (149.60 ± 38.70 g/L) in [26] compared to other studies with unconstrained sampling time.

BRIX was found to be lower for first and second parity cows than for those in later parities (Fig. 1) and there was variability across the nine herds in terms of colostrum Ig and BRIX, indicating that colostrum composition differs according to environmental factors, i.e. due to farm management [12, 27]. Least squares means of IgG, IgA, and IgM of this experiment have been previously published [12] and the evolution of IgG across parities is similar to that of BRIX (Fig. 1), with cows at first and second lactation showing the lowest colostrum quality. Cordero Solórzano et al. [24] observed a similar trend for ELISA IgG measured in Swedish cows’ colostrum across parities. This author, as well as Costa et al. [12], hypothesized that this trend may be due to a greater and cumulative exposure to antigens in older cows.

As regards ${h}^{2}$ estimates, the null hypothesis of the log-likelihood ratio tests was accepted; the limited sample size impaired the statistical power of the study and did not allow additive genetic variance to be significant (P > 0.05; Table 1). In line with this, standard errors of ${\mathrm{r}}_{\mathrm{ag}}$ were often large, whereas the ${\mathrm{r}}_{\mathrm{p}}$ showed smaller uncertainty (Tables 2 and 3). Similar situations have been reported in other studies dealing with colostrum genetic parameters using datasets of a similar/smaller size compared to that used here [3, 24].

Overall, both colostral BRIX and Ig showed lower ${h}^{2}$ as compared to fat and protein content of milk [28–30]. Nevertheless, colostrum traits were more heritable than health traits [31] and related phenotypes, such as milk SCC [28–30]. Heritability estimates for colostral BRIX of 0.27 ± 0.09 and 0.31 ± 0.06 were obtained in Holstein cows in Greece [3] and various Swedish breeds [24], respectively, which overlap with our estimates (0.26 ± 0.14). The repeatability of BRIX (0.35 ± 0.04) was estimated for the first time by Cordero Solórzano et al. [24] using repeated measurements on cows in different parities. Authors explained the low repeatability with the fact that in cows there is a continuous acquisition of new antibodies during life, making the Ig concentration of serum and colostrum across parities variable [24].

Genetic characteristics of IgG, IgA, and IgM concentration have been investigated by few authors in dam colostrum and/or calf blood. As an example, a Canadian research group took blood samples from 156 Holstein calves (progeny of 15 bulls) in the first 24–36 h after birth to determine Ig isotype concentration through RID and explore the genetic variability of the traits [32]. The authors estimated ${h}^{2}$ of 0.18 ± 0.25 for IgG, 0.12 ± 0.23 for IgA, and 0.26 ± 0.20 for IgM using a sire model. de Klerk et al. [33] harvested blood and milk from lactating Dutch cows and measured the natural antibody level, i.e. Ig of a self-renewing origin. In that study, ${h}^{2}$ was 0.15 ± 0.05 and 0.25 ± 0.06 for serum natural antibodies isotypes IgG and IgM, respectively. The IgG and IgM of milk were characterized by a lower ${h}^{2}$, i.e. 0.08 ± 0.03 and 0.23 ± 0.05, respectively [33]. Natural antibodies measured in milk and blood were strongly genetically correlated in the case of both IgG (0.81 ± 0.18) and IgM (0.79 ± 0.09) [33], suggesting that milk natural antibodies could be used as an indicator for genetic improvement of dairy cows’ health response. In primiparous Charolais cattle, the ${h}^{2}$ of colostral IgG determined with RID was 0.28 ± 0.14, whereas for the calf’s serum IgG concentration the estimated ${h}^{2}$ was 0.36 ± 0.18 [34].

According to the literature, the r_p_ between BRIX and IgG in mammals’ colostrum is strong and positive, thus indirect measurement using refractometers is considered sufficient for an estimation of IgG on dairy farms. In the present study, the r_p_ of BRIX with the three Ig averaged 0.65, with the r_p_ with IgG being the strongest (Table 2). To the best of our knowledge, only Cordero Solórzano et al. [24] estimated correlations between BRIX and ELISA-measured IgG in cow colostrum, reporting r_p_ of 0.70 ± 0.02 and r_ag_ of 0.68 ± 0.14.

Based on the selection index theory, the r_ag_ between the proxy (e.g. BRIX) and the target trait (e.g. RID IgG) is essential to assess the expected indirect response to selection. However, to evaluate if either a favorable or an unfavorable association exists between colostral quality indicators and protein-related traits, correlations with PC_C_, PC_M_, and PY_M_ were separately estimated for BRIX and IgG (Table 3). This is required, as the official selection index of Italian Holstein places emphasis on both protein content and yield [35]. In this selection index, protein yield receives the greatest weighting (33% of relative emphasis). As expected, both BRIX and IgG were strongly correlated with PC_C_. On the contrary, BRIX and IgG were neither genetically nor phenotypically associated with PC_M_ and PY_M_ in early lactation (5 to 75 days in milk). To our knowledge, this is the second study estimating r_p_ and r_ag_ between colostrum quality and milk traits, following a recent study conducted in Sweden [24]. In both cases, as previously indicated, phenotypic records from only a few hundred cows were available, resulting in large standard errors of the genetic parameters and uncertain interpretation. As an example, milk fat content was negatively genetically correlated with BRIX (− 0.15 ± 0.13), which is the opposite to that reported for milk fat and IgG (0.15 ± 0.21) [24]. Similarly, the r_ag_ between BRIX and milk yield (0.25 ± 0.17) and between IgG and milk yield (− 0.02 ± 0.28) are both close to/overlapping with 0. When, instead, milk protein content is considered, the authors estimated r_ag_ of 0.00 ± 0.12 with BRIX and 0.23 ± 0.20 with IgG [24]. To validate these findings, it is recommended that more research on the associations between colostrum and milk is conducted in dairy cattle with larger datasets.

The ${\mathrm{r}}_{\mathrm{ag}}$ of BRIX with PC_C_ close to 1 suggests that—at least genetically—they can indeed be considered as the same trait in colostrum sampled within 6 h from calving. The same applies to BRIX with IgG and PC_C_ with IgG, which have ${\mathrm{r}}_{\mathrm{ag}}$ close to 1 (Table 3). However, at the laboratory level, refractometers (${\mathrm{r}}_{\mathrm{p}}$ = 0.85 ± 0.08) cannot provide a prediction of IgG concentration that is sufficiently accurate for punctual determinations.

The thresholds used for the ROC analyses were in agreement with the general recommendations for calf feeding; in fact, according to the literature, good-quality colostrum should not provide less than 50 g/L of IgG [1, 2, 12]. The output of the ROC analysis summarized in Table 4, particularly the two AUC values (both ≥ 0.90), suggests that BRIX has an excellent diagnostic accuracy and is a precise indicator [22]. In fact, the diagnostic accuracy decreases as the AUC reduces: > 0.90 (excellent diagnostic accuracy), 0.89 to 0.80 (very good diagnostic accuracy), 0.79 to 0.70 (good diagnostic accuracy), 0.69 to 0.60 (sufficient diagnostic accuracy), 0.59 to 0.50 (bad diagnostic accuracy), and < 0.50 (insufficient diagnostic accuracy) [22]. In studies on dairy cattle [6, 9], a BRIX ≥ 22% was proposed for identification of good-quality colostrum; in general, small dissimilarities among studies may be related to different reference analyses, the type of refractometer used, breed, number of animals and herds involved, and, above all, time window and protocol for colostrum sampling.

An optimal transfer of passive immunity is reached when colostral IgG concentration is greater than 90 g/L [1]. In the presence of optimal volume intake, such concentrations should eliminate or limit cases of failure of passive transfer, which is defined as a serum IgG concentration below 12 g/L 12 h after birth [36]. Best practices in colostrum management and calf feeding rely on the newly defined ‘4Q’ rule: quality, quickness, quantity, and quota [13]. Storing colostrum in excess, in fact, is important to cover critical situations in which the cow produces either an insufficient amount of colostrum, low-quality colostrum, or does not yield colostrum at all [37]. Therefore, as with milk, the relationship between quantity and quality of colostrum must also be considered when assessing proposed breeding strategies. In addition, there is no consensus in the literature on the relationship between overall colostrum volume and quality. Given this, it is desirable that future studies investigate the ${\mathrm{r}}_{\mathrm{ag}}$ between colostral quality traits (BRIX or IgG) and the volume yielded by the cow at first milking, preferably measured within the first 6 h post calving. Moreover, it would also be advisable to investigate other factors with a potential role in colostrum quality and yield, such as dry period management and length, dam’s body condition score and age at first calving, and the exact colostrum sampling time (hours) post calving. As widely discussed in previous studies [12, 13], incorporation of exhaustive and complete data would improve the robustness of future colostrum-based experimental trials and designs.

While RID analyses result in a punctual concentration of IgG (expressed as g/L), the refractive index is expressed on a BRIX scale (%) and can be used as a proxy for colostrum quality [38], particularly when economical investments for colostrum analysis are not feasible or not pursued by either the farmers or the dairy organizations. Refractometers are, in fact, commercially available, easy to handle, cheap, and have deeply contributed to the improvement of colostrum management in dairy cattle [38]. In future applications, either BRIX or infrared predictions of IgG could be helpful for organizations in charge of genetic and genomic evaluations, allowing the definition of indexes specifically intended for colostrum quality improvement [13–15].

Finally, the refractometer was routinely used on just one out of the nine commercial farms involved in the present study, which indicates that opting for either BRIX or infrared-predicted IgG depends upon the farmers’ willingness to collaborate with researchers and their perception of the issue.

## Conclusions

To understand if refractometers are able to provide reliable colostrum quality data, the ${\mathrm{r}}_{\mathrm{ag}}$ and ${\mathrm{r}}_{\mathrm{p}}$ of BRIX with RID colostrum Ig concentrations were estimated in Holstein cows. In addition, the diagnostic accuracy of BRIX was evaluated through a ROC analysis. Colostrum BRIX had a strong positive genetic correlation with both IgG and IgM concentrations and exhibited an exploitable genetic variation. The refractometer was characterized by an excellent diagnostic accuracy for IgG concentration. Moreover, the correlation with milk protein content and yield in early lactation was estimated to be close to zero, indicating that selection would not impact current official breeding objectives. Our results indicate that the use of refractometers is advisable on equipped and well managed dairy farms to provide a proxy for colostral Ig isotype concentration. The availability of phenotypes at the population level would be beneficial for when and if breeders and/or breeders’ associations will consider that it is important to enhance colostrum quality through genetic selection. To promptly start selective breeding, it is crucial to effectively promote routine data recording as soon as possible, either via refractometers or infrared spectroscopy.

## Data Availability

The datasets generated and/or analysed during the current study are not publicly available but are available from the corresponding author on reasonable request.
